# Understanding Referral Patterns for Bone Mineral Density Testing among Family Physicians: A Qualitative Descriptive Study

**DOI:** 10.1155/2016/2937426

**Published:** 2016-01-19

**Authors:** Sarah E. P. Munce, Sonya Allin, Leslie Carlin, Joanna Sale, Gillian Hawker, Sandra Kim, Debra A. Butt, Irene Polidoulis, Karen Tu, Susan B. Jaglal

**Affiliations:** ^1^Department of Physical Therapy, University of Toronto, Toronto, ON, Canada M5G 1V7; ^2^Toronto Rehabilitation Institute, University Health Network, Toronto, ON, Canada M5G 2A2; ^3^Mobility Program Clinical Research Unit, Li Ka Shing Knowledge Institute, St. Michael's Hospital, Toronto, ON, Canada M5B 1W8; ^4^Institute of Health Policy, Management & Evaluation, University of Toronto, Toronto, ON, Canada M5T 3M6; ^5^Department of Medicine, University of Toronto, Toronto, ON, Canada M5S 1A8; ^6^Institute of Clinical Evaluative Sciences, Toronto, ON, Canada M4N 3M5; ^7^Women's College Research Institute, Toronto, ON, Canada M5S 1B2; ^8^The Scarborough Hospital, Scarborough, ON, Canada M1P 2V5; ^9^Department of Family and Community Medicine, University of Toronto, Toronto, ON, Canada M5G 1V7

## Abstract

*Introduction*. Evidence of inappropriate bone mineral density (BMD) testing has been identified in terms of overtesting in low risk women and undertesting among patients at high risk. In light of these phenomena, the objective of this study was to understand the referral patterns for BMD testing among Ontario's family physicians (FPs).* Methods*. A qualitative descriptive approach was adopted. Twenty-two FPs took part in a semi-structured interview lasting approximately 30 minutes. An inductive thematic analysis was performed on the transcribed data in order to understand the referral patterns for BMD testing.* Results*. We identified a lack of clarity about screening for osteoporosis with a tendency for baseline BMD testing in healthy, postmenopausal women and a lack of clarity on the appropriate age for screening for men in particular. A lack of clarity on appropriate intervals for follow-up testing was also described.* Conclusions*. These findings lend support to what has been documented at the population level suggesting a tendency among FPs to refer menopausal women (at low risk). Emphasis on referral of high-risk groups as well as men and further clarification and education on the appropriate intervals for follow-up testing is warranted.

## 1. Introduction

A bone mineral density (BMD) test is used to make a diagnosis of and screen for osteoporosis and to inform assessments of fracture risk. Clinical practice guidelines (CPGs) in Canada currently recommend BMD testing in specific at-risk populations, including testing to screen for osteoporosis in patients over 65 years of age or who experience a fragility fracture after the age of 40 [1, 2]. However, evidence of inappropriate testing has been identified in terms of overtesting in low risk women [3, 4] translating into unnecessary costs to the health care system and harm to patients through overdiagnosis and overtreatment [5]. In contrast, our research team has previously reported undertesting among high-risk patients at a population level [4]. In Canada and the US, about 50% of women over 65 years of age and 81% of men have not had a BMD test [6–8]. In a systematic review of practice patterns in the management of osteoporosis after fragility fracture, BMD testing was performed in less than 15% of patients with recent fractures in 15 of 23 studies [6]. Thus, a significant care gap exists between current guidelines and Canadian testing patterns.

One potential explanation for this care gap is the multiple sources of recommendations for BMD testing which do not always agree with the 2010 Canadian CPGs for the diagnosis and management of osteoporosis [1]. A recent survey of screening for and treatment of osteoporosis identified 24 different sets of published clinical guidelines for BMD testing [9]. In Ontario, the situation is further confounded by the fact that the current physician fee schedule still refers to the 2002 Canadian CPGs for the diagnosis and management of osteoporosis [10] which do not emphasize fracture risk or provide clear guidance for referral of high fracture risk patients even though reimbursement policies were changed in 2008 [11]. In light of the described care gap and these inconsistencies, the objective of the current study was to understand the referral patterns for bone mineral density (BMD) testing among Ontario's family physicians (FPs), as FPs currently refer the majority of Canadian BMD tests [12].

## 2. Methods

### 2.1. Design/Approach

We conducted in-depth semi-structured interviews by telephone. A qualitative descriptive approach as described by Sandelowski [13, 14] informed the design, collection, and analysis of data. This approach is well suited for researching topics about which little is known and yielding practical answers of relevance to health care practitioners [13, 14].

### 2.2. Recruitment

FPs were initially recruited through an event on osteoporosis held by the Ontario College of FPs. Snowball sampling was performed following the initial sampling whereby participants were asked to provide the name of a colleague who might be interested in participating. Eligible FPs were English speaking, in active family practice in Ontario, and had experience ordering and receiving results of BMD tests for patients. Participants were recruited between November 2011 and June 2012. Recruitment ceased as analysis of the data approached data saturation, the point where successive interviews did not generate novel responses or themes [15]. Research ethics approval was obtained from Women's College Hospital (Protocol Reference #2008-0064-E). All participants provided informed consent.

### 2.3. Data Collection

Each participant took part in a semi-structured interview lasting approximately 30 minutes. Interviews were conducted by two of the team members (Sarah E. P. Munce and Leslie Carlin). The interview guide consisted of semi-structured open-ended questions (e.g.,* For what reasons are you likely to refer a patient for a BMD test*?) and was pilot-tested. Probes were used during interviews to explore issues in greater depth and verify the interviewer's understanding of the information being collected [15]. All interviews were digitally recorded and transcribed verbatim for data analysis.

### 2.4. Data Analysis

An inductive thematic analysis was performed on the transcribed data in order to understand the reasons for referral for BMD testing [16]. A subset of interview transcripts was initially coded by the first author (Sarah E. P. Munce). Two other researchers (Sonya Allin and Leslie Carlin) independently coded this subset and met to compare assigned codes. A coding framework was then developed and applied to the remaining transcripts. To facilitate the organization and analysis of the qualitative data, transcripts and reflective notes from the interviews were entered into NVivo 9 [17]. Codes were then clustered into groups or categories and predominant themes were identified. To maximize credibility and trustworthiness, three members of the research team (Sarah E. P. Munce, Sonya Allin, and Leslie Carlin) discussed the developing analysis and identified new themes. Together, the researchers explored various thematic maps until consensus was reached and theme labels were agreed upon. The first author then analyzed the remaining data. Quotations from interview participants are presented in Tables 1–3.

## 3. Results

### 3.1. Demographics

Twenty-two FPs participated in the interviews. Twelve of the participants were male and five of the participants were from the Ontario College of FPs' event. All of the participants had practices located in urban or suburban Ontario; 15 participants indicated that they were a member of a group practice. The mean roster size of the physicians was 1208 and participants reported reviewing an average of four BMD reports weekly.

### 3.2. Overview of Themes: Uncertainty about Screening and Follow-Up Testing

FPs were clear and accurate in their interpretation of guidelines for BMD testing among the following patient groups: individuals who had a (recent) fracture, those who had used glucocorticoids, or those who had secondary causes of osteoporosis. Almost half (*n* = 10/22) of the FPs also indicated that they ordered a follow-up BMD test to monitor changes in bone density during the course of pharmacologic treatment. There was uncertainty about screening postmenopausal women in the absence of other clinical risk factors, seniors, men, and follow-up testing intervals.

#### 3.2.1. Screening Postmenopausal Women in the Absence of Other Clinical Risk Factors

Many (*n* = 16/22) of the FPs reported that they ordered what they regarded as a “baseline” BMD test for postmenopausal patients in the absence of other clinical risk factors. Sometimes this was part of a “well woman” annual visit in which the patient would also have a mammogram. These particular BMD tests sometimes coincided with the patient herself requesting a BMD test (see Table 1 for quotes).

#### 3.2.2. Screening Seniors, Men in Particular

Almost half (*n* = 10/22) of the FPs were unclear about the appropriate age for baseline BMD screening for osteoporosis, particularly in men. The perceived appropriate age for screening in men ranged from “*an over 50*” (Interview 8) to “*over 60 or 65*” (Interview 6) to “*I'm not in the routine of doing everybody over 65*” (Interview 10). At the same time, FPs explained that they had made a recent, concerted effort to make referrals for their male patients. The reasons for this effort were unknown/unclear (see Table 2 for quotes).

### 3.3. Uncertainty about Bone Mineral Density Follow-Up Testing Intervals

Finally, FPs indicated that they would order a BMD test for the purposes of monitoring, especially for examining the impact of pharmacotherapy on bone density (*n* = 10/22). However, some of the FPs indicated an associated uncertainty about the appropriate timing for this testing with some of the physicians indicating that this stemmed from a lack of evidence on appropriate interval(s) for this testing. FPs also indicated that the intervals for (subsequent) testing varied depending on whether or not a patient's BMD was stable or improving (i.e., the intervals increased) or worsening (i.e., the intervals decreased). The expectations for BMD improvements were often inaccurate: one FP, for example, stated that gains in hip bone mass in excess of 5% were to be expected in response to treatment after one year (Interview 5). Several FPs expressed a desire for more concrete guidance on intervals related to specific clinical scenarios, such as duration of treatment or the use of drug holidays (see Table 3 for quotes).

## 4. Discussion

The current study aimed to understand the referral patterns for BMD testing among FPs in Ontario, representing the most populous province in Canada. Overall, we identified a lack of clarity about screening for osteoporosis with a tendency for baseline BMD testing in healthy, postmenopausal women and a lack of clarity on the appropriate age for screening for men in particular. A lack of clarity on appropriate intervals for follow-up testing was also described.

### 4.1. Uncertainty about Screening for Osteoporosis

In our study, several FPs recommended baseline screening for postmenopausal women in the absence of other risk factors, which is consistent with our previous research indicating overtesting (i.e., unnecessary testing) in low risk populations [3]. In addition, data from our interviews indicated that ambiguity regarding age as a risk factor for fracture may contribute to underscreening of at-risk individuals. Again, this is consistent with North American evidence demonstrating that only about 50% of senior women (i.e., 65 years of age and over) have ever had a BMD test [6]. Similarly, Lim and colleagues [18] determined that only 11.3% of US men over the age of 70 in a primary care setting had a BMD test. It is notable that, in the current study, some FPs indicated specifically that they had made a recent effort to refer male patients for BMD testing.

A review of CPGs for osteoporosis screening found that only 6 of the 11 guidelines recommended universal screening for women over the age of 65 [9]. While all of the guidelines endorsed screening postmenopausal women under the age of 65 who had an additional risk factor, there was no agreement on which risk factors should be considered in this regard. Furthermore, only 4 of the guidelines contained any explicit recommendations regarding screening for men [9]. Inconsistent CPGs surrounding screening for osteoporosis exist within Canada; while the 2010 Canadian CPGs for the diagnosis and management of osteoporosis recommend universal screening for all patients over age 65 [1], recent recommendations from the College of Family Physicians of Canada Choosing Wisely initiative now recommend screening of women over the age of 65 and men over the age of 70 [19].

### 4.2. Uncertainty about Appropriate Follow-Up Intervals for Bone Mineral Density Testing

Most of the FPs in the current study recognized there to be no “one size fits all” rule for follow-up testing in their patients. Several indicated that they employed different repeat testing heuristics for different categories of patients. The categories identified during the interviews included those with relatively stable BMD, those initiating treatment, and those with rapidly declining BMD. The specific heuristics used to monitor each of these categories varied widely as did the definition of “effective response” to treatment. One FP, for example, stated that improvement in hip BMD in excess of 5% was to be expected in response to treatment after one year; evidence from clinical trials suggest treatments increase BMD 1% to 6% after 3 years, on average [20]. While FPs generally indicated that they ordered follow-up BMD tests to inform pharmacotherapy management decisions, several expressed a desire for more concrete guidance on intervals related to specific clinical scenarios, such as duration of treatment or the use of drug holidays.

Uncertainty around testing intervals, like uncertainty regarding screening, reflects a lack of consensus in published evidence. The US Preventive Task Force (USPSTF) specifically identified intervals for repeat testing as an area lacking clarity, particularly for those with normal BMD on baseline testing [21]. In 2012, a study that followed BMD changes in almost 5000 women 67 years of age or older determined that, on average, it took more than 15 years for 10% of those with “normal” BMD to transition to osteoporosis [22]. While repeat intervals for those with normal BMD are unclear, repeat intervals for those with low BMD and/or changing BMD are equally unclear. Furthermore, measurable BMD changes for individuals on therapy may take several years to manifest. For example, in a secondary analysis of trial data comparing the effects of alendronate and placebo on over 6000 postmenopausal women with low BMDs, the authors concluded that monitoring BMD in postmenopausal women in the first three years after starting treatment with a bisphosphonate is unnecessary and may be misleading [23].

In Canada, the 2010 CPGs for the diagnosis and management of osteoporosis [1] suggest that, for patients undergoing treatment, repeat testing “should be performed after one to three years” but that the testing interval can be increased should therapy be determined to have an “effective response”; however, limited details are provided as to the meaning of “effective response” to treatment. Similarly, the National Osteoporosis Foundation (NOF) [24], the International Society for Clinical Densitometry [25], and the USPSTF [21] recommend BMD testing one to two years after starting or changing therapy [24, 25]. All of the above guidelines endorse repeat testing as early as one year which is inconsistent with testing intervals currently supported by US Medicare [26], for example, or endorsed by the Canadian Rheumatology Association. For example, the Canadian Rheumatology Association's current recommendations for the Choosing Wisely Canada campaign suggest refraining from testing at intervals less than two years “in most clinical settings,” as this amount of time is required to “reliably measure a change in BMD” [27].

### 4.3. Clinical Implications

(1) Family physicians require better guidance regarding screening for osteoporosis, particularly surrounding the risk factors that warrant further investigation. Several of the interviewed FPs associated the onset of menopause with risk of osteoporosis. While age was widely recognized as a risk factor for osteoporosis, the specific age necessitating screening by BMD testing was not clear, particularly for men. Interventions that clarify risk factors that warrant screening with BMD are in need.

(2) Family physicians require better guidance regarding testing intervals that are stratified by key clinical scenarios, for example, those initiating or changing treatment, those contemplating or on drug holidays, and those with active risk factors for bone loss (e.g., ongoing glucocorticoids or secondary causes). The definition of “effective response” to treatment needs to be better communicated by guidelines, along with the duration of time that is needed to reliably gauge effectiveness.

### 4.4. Limitations

This study acknowledges some limitations. Some of the participants were initially recruited via an educational event related to osteoporosis. As such, it is possible that they had an active interest in the area of osteoporosis and a higher degree of knowledge related to the subject. If this were the case, it is likely that gaps in knowledge and practice among a general population of FPs would likely be even larger. Further, the interviewed participants were all from urban or suburban areas in Ontario. It is unknown whether differences in knowledge and practice would have been observed between the physicians interviewed in the current study and those in rural practices or other provinces. Lastly, it is important to acknowledge the limitations of BMD testing, as many fragility fractures occur in individuals without any densitometric osteoporosis. It is possible that this knowledge of its limitation impacts referral patterns, although this was not discussed with the FPs in the current study.

### 4.5. Future Research and Conclusion

As suggested in the 2010 Canadian CPGs for the diagnosis and management of osteoporosis [1], point-of-care tools and other targeted strategies are recommended to support the implementation of osteoporosis guidelines in clinical practice. As such, future research should involve the development, implementation, and evaluation of such tools in order to assist FPs in making more appropriate referrals for BMD-based screening and repeat exams. Recently, British Columbia, Manitoba, and Nova Scotia have developed standardized requisitions for BMD testing which communicate guidelines and act as point-of-care decision aids. However, the effect of these requisitions on testing patterns has yet to be determined.

Findings from this qualitative study lend support to what has been documented at the population level as well as our own recent research suggesting a tendency among FPs to refer menopausal women (at low risk). Consistency among CPGs, emphasis on referral of high-risk groups as well as men, and further clarification and education on the appropriate intervals for follow-up testing are warranted. This may be accomplished via the development and implementation of a standardized requisition tool to assist FPs in making appropriate referrals for BMD testing.

## Figures and Tables

**Table 1:** Screening postmenopausal women in the absence of other clinical risk factors.

Interview 10	…* often I'm doing [BMD tests] at menopause time in a woman's life when things sort of come up. I get a baseline maybe at menopause *

Interview 13	*Okay, [a referral] yesterday, a woman in for her physical. She is newly menopausal. It's been years since her last period, 50 years old, smoker … That particular case did not have any other risk factors*

Interview 20	*Then occasionally [I make a referral] when women say “I need a bone density, don't I” and they argue with me and they say their gynecologist does it and they have no reason to but I get tired of arguing*

Interview 21	*I try to [make referrals] based on the osteoporosis guidelines, anybody over the age of 65 male or female I order it, women who are post- menopausal and wanting to consider just a baseline bone density and *…* other people who have other risk factors*

Interview 22	*I guess right now [I refer] mainly for screening postmenopausal women*

What the 2010 Canadian CPGs for the diagnosis and management of osteoporosis indicate.	**(i) Indications for baseline BMD testing include all women and men age ≥65**
	**(ii) Ordering a BMD test in women younger than 65 years of age as a baseline test because they are menopausal is not indicated in the absence of other risk factors**

**Table 2:** Uncertainty about screening seniors, men in particular.

Interview 1	*I do have some men in my practice and I do try to be alert to the fact that men need to be checked too, particularly if it's a thin man or somewhat frail. You're kind of more prompted. If I get a big burly guy who's 70, I have to admit probably I don't check their bones all that often*

Interview 3	*Men I'll do it *…* if they're 55 years of age or older *

Interview 4	*It wasn't really indicated before *…* I don't know how strong the evidence is for men for screening*

Interview 17	*Because I work in a teaching practice, my residents are very devoted to guidelines. A lot of them are driven by the more recent guidelines … which is I think reasonable for women but I think problematic for men*

Interview 22	*The gentleman had never had one done and he's in his 70s. So I ordered it because of that *

What the 2010 Canadian CPGs for the diagnosis and management of osteoporosis indicate.	**(i) Indications for baseline BMD testing include all women and men age ≥65**

**Table 3:** Uncertainty about bone mineral density testing intervals.

Interview 2	*Then I'll see when they've had their last bone density *…* and I'll say “well you just had one two years ago, you're on treatment, it was stable from the year before, and I don't think you need one.” Well why shouldn't we do one? Then I'll say “well is it going to change your management in any way” *…* What does usually happen is that they usually win *

Interview 3	*Her last BMD was a year and a half ago. I sent her for another one just to monitor her therapy. I mean we don't really have any clear guidelines as to what we should be doing for patients that are on therapy. So we send them for a BMD to look at change.* …* This usually comes up when we have our annual physical *

Interview 5	*You order a bone density a year later and you want to see whether or not the treatment has worked. To be significant gain for the lumbar spine, it's 3% and for the hip it's 5% to 6%. If they're not getting those gains within a year, I might decide to change … the method in which it's administered *

Interview 6	*She had one done about three years ago and it showed osteopenia. We just opted for calcium and vitamin D. She was in the office the other day with her new BMD that it showed it was pretty stable. So we're still continuing with the same management *

Interview 7	*If *…* a patient was started on something, then I usually repeat [the BMD test] after a year. If the BMD is stable then I'll repeat it maybe after three years*

Interview 8	*And follow up, the first time I put them on medication, I'll follow up in two years. The ones that are normal, it's every five years *

What the 2010 Canadian CPGs for the diagnosis and management of osteoporosis indicate.	**(i) “For patients who are undergoing treatment, repeat measurement of BMD should initially be performed after one to three years; the testing interval can be increased once therapy is shown to be effective. If BMD has improved or remains unchanged, the patient is considered to have had a good response to therapy”**
	**(ii) The definition of good response to treatment needs to be better communicated by guidelines, along with the duration of time that is needed to reliably gauge effectiveness **
